# Mapping cognitive deficits in cancer patients after chemotherapy: An Activation Likelihood Estimation meta-analysis of task-related fMRI studies

**DOI:** 10.1007/s11682-022-00655-4

**Published:** 2022-04-02

**Authors:** Jacqueline B. Saward, Elizabeth G. Ellis, Annalee L. Cobden, Karen Caeyenberghs

**Affiliations:** Cognitive Neuroscience Unit, School of Psychology, Deakin University, Melbourne Burwood Campus, 221 Burwood Highway, Burwood, VIC 3125 Australia

**Keywords:** Cancer, Cognitive deficits, Chemotherapy, Functional MRI, Activation Likelihood Estimation

## Abstract

Recent neuroimaging studies have reported alterations in brain activation during cognitive tasks in cancer patients who have undergone chemotherapy treatment. However, the location of these altered brain activation patterns after chemotherapy varies considerably across studies. The aim of the present meta-analysis was to quantitatively synthesise this body of evidence using Activation Likelihood Estimation to identify reliable regions of altered brain activation in cancer patients treated with chemotherapy, compared to healthy controls and no chemotherapy controls. Our systematic search identified 12 studies that adopted task-related fMRI on non-central nervous system cancer patients who received chemotherapy relative to controls. All studies were included in the analyses and were grouped into four contrasts. Cancer patients treated with chemotherapy showed reduced activation in the left superior parietal lobe/precuneus (family-wise error corrected *p* < .05) compared to no chemotherapy controls. No significant clusters were found in three of our contrasts. The majority of studies did not support an association between altered brain activation and cognitive performance after chemotherapy. Findings point towards a possible chemotherapy-induced alteration, which could inform targeted treatment strategies. With continued work in this field using homogenous task-related protocols and cancer populations, fMRI may be used as a biomarker of cognitive deficits in the future.

## Introduction

Cancer is a major cause of death and illness worldwide, with an estimated 19.3 million new cases and 10 million deaths in 2020 (Sung et al., 2021). While chemotherapy has improved cancer survival rates, it is often associated with adverse treatment-related side-effects, including cognitive deficits (Janelsins et al., 2014). Self-reported measures and computerised neuropsychological tests have identified cognitive deficits in cancer survivors who have undergone chemotherapy regimens, often dubbed “chemobrain” or “chemofog” (for a review, see Hutchinson et al., 2012). The most prominently affected domains in cognitive functioning include impairments in processing speed, memory and executive functions (Ahles et al., 2002; Jansen et al., 2011; Wefel et al., 2010). These symptoms are prevalent across all types of cancer, including those which manifest outside the central nervous system (CNS). Cognitive deficits can have pervasive impacts on cancer survivors’ quality of life, interfering with their capacity to accomplish daily tasks (e.g., cooking and driving), as well as their interpersonal relationships and occupational performance (Boykoff et al., 2009; Henderson et al., 2019; Myers, 2012). Therefore, it is important to examine the biological underpinnings of cognitive deficits following chemotherapy to inform treatment strategies.

Over the past two decades, functional magnetic resonance imaging (fMRI) studies have yielded novel insights into the neural substrates of cognitive deficits in cancer patients by investigating aspects of brain function (for a review, see Sousa et al., 2020). Task-related fMRI has been utilised to detect neural abnormalities in cancer patients treated with chemotherapy (CTx+) by comparing brain activation patterns during cognitive tasks with healthy controls (HC) and/or cancer patients who received surgery or adjuvant treatments but not chemotherapy (CTx−; no chemotherapy controls). Utilising this MRI technique for the first time, a case study presented in Ferguson et al. (2007) compared monozygotic twins, reporting increased frontal and parietal activation during a working memory task in a twin treated with chemotherapy compared to the healthy twin. Since this initial contribution, several cohort task-related fMRI studies have been undertaken. In one of the first group studies, Kesler et al. (2009) reported both increased activation in multiple brain regions during a memory recall task and decreased activation in the prefrontal cortex during a memory encoding task in CTx+ patients relative to HC. More recent studies have observed similar findings to Ferguson et al. (2007), with increased activation found in frontal regions during working memory and episodic memory tasks in CTx+ patients compared to controls (McDonald et al., 2012; Pergolizzi et al., 2019). Conversely, other studies have shown reduced activation in frontal and parietal regions (Correa et al., 2017; López Zunini et al., 2013; Stouten-Kemperman et al., 2015) and subcortical regions (e.g., hippocampus and amygdala; de Ruiter et al., 2011; Vardy et al., 2019; Wang et al., 2016) in CTx+ patients relative to controls. Despite these notable findings, the localisation and direction of altered brain activation patterns are mixed across studies, rendering interpretation difficult.

Among popular theories, brain regions of decreased activation in CTx+ patients compared to controls, have been interpreted as the result of chemotherapy-induced damage (Correa et al., 2017; Kesler et al., 2011). Animal studies have demonstrated that non-CNS chemotherapeutic agents can have neurotoxic effects on the structure and function of normal cells in the nervous system (Briones & Woods, 2011; Christie et al., 2012; Yan et al., 2015). Contrarily, increased brain activation in CTx+ patients compared to controls, has been suggested as a compensatory mechanism for dysfunction in task-relevant brain regions, with the recruitment of additional brain regions required to reach the same level of performance (Ferguson et al., 2007; McDonald et al., 2012; Menning et al., 2017). With the identification of consistent altered brain activation patterns in CTx+ patients, present understandings may be strengthened.

To date, a number of scoping and systematic reviews have performed a *qualitative* comparison on brain activation patterns in cancer survivors across several studies, highlighting diffuse alterations in frontal and parietal regions after chemotherapy (Andryszak et al., 2017; de Ruiter & Schagen, 2013; Li & Caeyenberghs, 2018; Pomykala et al., 2013; Scherling & Smith, 2013; Simó et al., 2013; Sousa et al., 2020). Aside from these reviews, only one *quantitative* study (Kesler, 2014) has been published so far. In this study by Kesler (2014) a meta-analytical technique known as Activation Likelihood Estimation (ALE) was used to synthesise data across six fMRI studies (Conroy, McDonald, Smith, et al., 2013a; de Ruiter et al., 2011; Kesler et al., 2009; Kesler et al., 2011; López Zunini et al., 2013; McDonald et al., 2012), revealing consistently reduced activation in brain regions involved in the default mode network (e.g., the precuneus and medial frontal gyrus). However, this study was limited by not reporting the group contrasts and methodological procedures used in their ALE analysis.

In the present study, we will integrate the most-recent fMRI data to identify *robust* patterns across studies, utilising ALE. The ALE approach has successfully been employed to map neural correlates of symptoms in a wide array of neurological and psychiatric populations, including patients with Parkinson’s disease (Santangelo et al., 2019), traumatic brain injury (Cook et al., 2020) and major depressive disorders (Zhang et al., 2016). During fMRI, neural locations of peak activation evoked by performance on functional tasks are recorded in stereotaxic coordinates (Fuelscher et al., 2018). An ALE meta-analysis aggregates these significant activation coordinates from multiple neuroimaging studies to identify voxel-wise regions of spatial convergence (Eickhoff et al., 2009; Eickhoff et al., 2012). The resulting map incorporates commonly activated neuroanatomical regions across individual studies (Eickhoff et al., 2012). In other words, ALE enables a way of determining how likely a neural location is to be involved in a symptom based on several studies (Eickhoff et al., 2012; Fuelscher et al., 2018). Overall, ALE offers a powerful method for aggregating data from neuroimaging studies as it attempts to minimise study-specific noise of small sample sizes and various experimental tasks (Eickhoff et al., 2009; Eickhoff et al., 2012). An ALE analysis will therefore provide much needed clarification into the potential neural mechanisms underlying cognitive deficits in cancer patients after chemotherapy.

### Aims and hypotheses

The aims of the present study are twofold. The primary aim is to conduct an ALE meta-analysis to identify reliable regions of altered brain activation in CTx+ patients across several task-related fMRI studies. Specifically, we will examine whether there are differences in brain activation patterns during cognitive tasks between non-CNS CTx+ patients, compared to HC and CTx− patients. It is hypothesised that ALE will reveal consistent alterations in brain activation (i.e., increased activation in frontal and parietal regions and decreased activation in subcortical regions) in CTx+ patients compared to control groups. Our secondary aim is to explore the association between altered brain activation and cognitive performance (i.e., using performance scores from a cognitive task performed as part of the behavioral test battery) in CTx+ patients. This may inform whether regions of altered brain activation patterns are reliable biomarkers of cognitive deficits experienced by cancer survivors. Mixed results have been found in the literature with some studies reporting a positive association (i.e., decreased brain activation associated with worse cognitive task performance; de Ruiter et al., 2011) and other studies revealing no significant association (Correa et al., 2017; Wang et al., 2016). It is expected that reduced brain activation will be related to diminished performance on cognitive tasks in CTx+ patients.

## Methods

### Systematic search

A systematic search of peer-reviewed literature published up to 30 July 2020 was conducted. Embase, PsycInfo and MEDLINE Complete databases were searched. The search string involved a combination of keywords including “fMRI”, “cancer”, “chemotherapy” and “cognitive dysfunction”, and their synonyms, based on a recent systematic review (Li & Caeyenberghs, 2018). See Appendix 1 Table 3 for the full search syntax. No publication date or language limiters were applied. Reference sections of eligible studies were inspected to identify additional articles of interest.

Studies were included in the analysis if they met the following criteria: (1) utilised task-related fMRI as the main neuroimaging modality; (2) participants performed a cognitive task during scanning; (3) reported coordinates of activation foci in either Montreal Neurological Institute (MNI) or Talairach reference space; (4) reported group comparisons between cancer patients treated with chemotherapy compared with healthy controls and/or no chemotherapy controls. Studies with only restricted regions of interest (ROI) analyses were excluded to ensure that the likelihood of activation was equal across the brain (Eickhoff et al., 2009; Muller et al., 2018). Articles that investigated cancer of the brain or central nervous system (CNS) were also omitted as neurosurgery and CNS-directed chemotherapy are known to induce neurocognitive impairments or brain alterations (Li & Caeyenberghs, 2018). Studies conducted in paediatric cancer populations (age < 18 years) and animal models were removed. Finally, conference abstracts, case studies and systematic reviews were excluded.

### Data extraction

We extracted and summarised data on clinical population characteristics from each study, including the age and gender of participants, cancer type, time since treatment, chemotherapy regimens and adjuvant treatments (see Appendix 2 Table 4). The steps for neuroimaging analyses of each study are detailed in Table 1, including group contrasts, sample size, activation foci, brain template, statistical thresholding and fMRI paradigm used. Finally, data was extracted from selected studies which performed correlation analyses between regions of altered brain activation patterns and cognitive performance in the chemotherapy-treated group, including the significance of the relationship, correlation coefficients, and the involved brain regions (Table 1).

**Table 1:** Overview of the studies included in the ALE analyses

Study	Contrast	*N*	Foci	Template	Statistical threshold	Cognitive domain (fMRI task)	Correlation analyses
Conroy, McDonald, Smith, et al. (2013a)	CTx+ < HC	47	2	MNI	*p* < .001 uncorrected	Working memory (Verbal n-back)	Tested correlation, but no significant association found
Correa et al. (2017)	CTx+ < HC	35	8	Talairach	*p* < .005 uncorrected	Attention and working memory (Visual n-back)	Tested correlation, but no significant association found
de Ruiter et al. (2011)	CTx+ < CTx− CTx+ > CTx−	31	10 1	MNI	*p* < .001 uncorrected	Executive function (Tower of London) and Episodic memory (Paired associates learning)	Positive association between DLPFC activation and executive function performance (*r* = .77, *p* < .001) and parahippocampal activation and episodic memory performance (*r* = .79, *p* < .001)
Kesler et al. (2009)	CTx+ < HC CTx+ > HC	28	1 1	MNI	*p* < .05 FDR corrected	Verbal memory (Encoding and Recall)	Tested correlation, but no significant association found
Kesler et al. (2011)	CTx+ < HC CTx+ < CTx−	39 40	3 1	MNI	*p* < .05 FDR corrected	Executive function (Wisconsin card sorting test)	Tested correlation, but no significant association found
López Zunini et al. (2013)	CTx+ < HC	42	4	MNI	*p* < .001 uncorrected	Verbal memory (Recall)	N/A
McDonald et al. (2012)	CTx+ < HC CTx+ > HC CTx+ > CTx−	31 28	1 1 3	MNI	*p* < .001 uncorrected	Working memory (Verbal n-back)	N/A
Menning et al. (2017)	CTx+ > CTx−	44	9	MNI	*p* < .05 FWE corrected	Executive function (Tower of London)	N/A
Pergolizzi et al. (2019)	CTx+ < HC CTx+ > HC	24/25	1 10	MNI	*p* < .05 FWE corrected	Episodic memory (Levels of processing)	N/A
Stouten-Kemperman et al. (2015)	CTx+ < CTx−	39	9	MNI	*p* < .001 uncorrected	Executive function (Tower of London) and Episodic memory (Paired associates learning)	Positive association between DLPFC activation and executive function performance (*t* = 4.13) and hippocampal activation and episodic memory performance (*t* = 4.54). No *p* value reported
Vardy et al. (2019)	CTx+ < CTx−	57	6	MNI	*p* < .001 uncorrected	Work memory (N-back)	N/A
Wang et al. (2016)	CTx+ < HC	29	1	Talairach	*p* < .05 corrected	Working memory (Visual n-back)	Tested correlation, but no significant association found

*Note.* CTx+ = cancer patients treated with chemotherapy; CTx− = no chemotherapy controls; HC = healthy controls; < = decreased brain activation; > = increased brain activation; *N* = sample size; MNI = Montreal Neurological Institute; FDR = false discovery rate; FWE = family-wise error; DLPFC = dorsolateral prefrontal cortex; N/A = no correlation analyses conducted

The parameters for an ALE analysis (i.e., sample size and activation foci) were manually imported into text files according to the type of group contrast and the direction of the contrast. Coordinates that were reported in Talairach space were transformed into MNI coordinates using the BioImage Suite tool (Lacadie et al., 2008). In studies that reported foci from two different paradigms (Correa et al., 2017; de Ruiter et al., 2011; Kesler et al., 2009; Kesler et al., 2011; Stouten-Kemperman et al., 2015), the foci were included as one experiment. However, for the study of Pergolizzi et al. (2019), results were imported as two experiments to account for the different number of participants that completed each paradigm. For longitudinal studies (López Zunini et al., 2013; McDonald et al., 2012; Menning et al., 2017; Pergolizzi et al., 2019), data was extracted from the last follow-up time-point. In studies that examined two cohorts of chemotherapy-treated patients, coordinates were selected from the groups of patients who received standard-dose chemotherapy (Stouten-Kemperman et al., 2015) and patients who reported cognitive symptoms (Vardy et al., 2019).

### Activation Likelihood Estimation

**Fig. 1:**
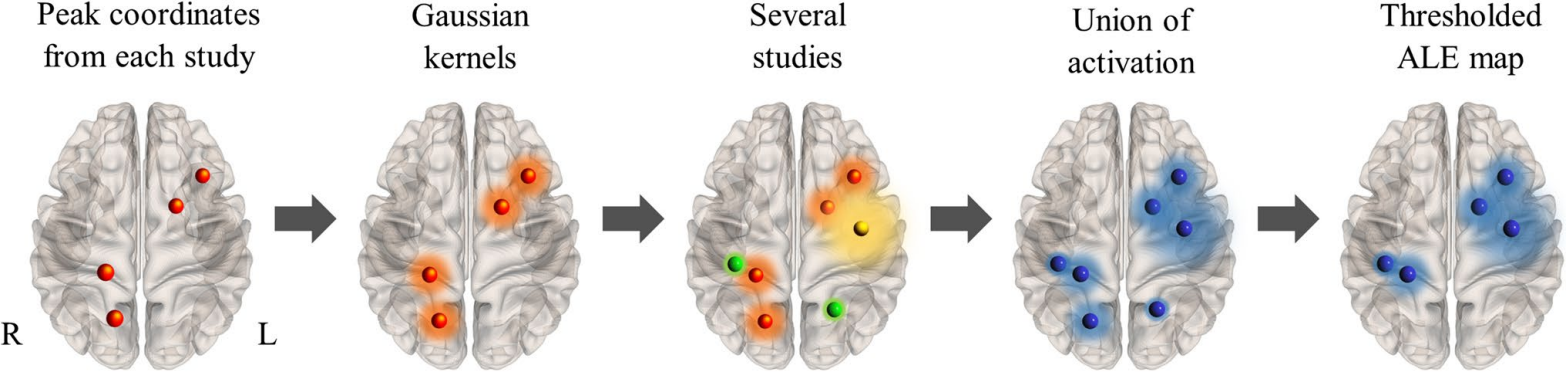
Overview of the ALE meta-analysis procedure. *Note.* R = right hemisphere; L = left hemisphere. Orange, green and yellow colours indicate activation foci obtained from the different studies. Brain surface models were created using the NeuroMarvl tool (https://immersive.erc.monash.edu/neuromarvl/)

To examine the first research question, we conducted four independent ALE analyses using Ginger ALE’s (version 2.3.6; http://brainmap.org) random effects algorithm (Eickhoff et al., 2009; Turkeltaub et al., 2012). The ALE technique aggregates *statistically significant* foci reported from multiple neuroimaging studies. In other words, contrasts of ALE meta-analyses only include studies that have reported coordinates of brain regions that revealed significant group differences in brain activation (i.e., significant increases or decreases in activation between CTx+ patients and controls). This allows us to identify the brain regions which are most commonly implicated in cognitive symptoms in cancer patients across studies (Acar et al., 2018; Eickhoff et al., 2009). Figure 1 depicts a step-by-step overview of the procedure. Firstly, peak coordinates reported from each neuroimaging study are individually mapped onto a standardised brain. Secondly, to account for spatial uncertainty, Gaussian kernels are applied around the foci, whereby the diameter of the kernel is determined by the study sample size (e.g., smaller studies have more spatial uncertainty and therefore we apply larger kernels; Acar et al., 2018). This process results in a series of modelled activation (MA) maps. Next, an ALE map is computed by calculating the union of these MA maps. In the final step, the ALE map is thresholded by testing the activation likelihood values at each voxel against a null distribution of random spatial associations among studies, to establish at which locations the convergence of foci is greater than can be expected by chance (Eickhoff et al., 2012).

We corrected for multiple comparisons utilising a cluster-level family-wise error (FWE) threshold of *p* < .05, with 1000 permutations, following an initial cluster-forming threshold of uncorrected *p* < .001. This thresholding procedure provides an appropriate balance between sensitivity and specificity and is in line with recommendations from Eickhoff et al. (2012). The resulting maps were overlaid onto a standard anatomical template in MNI space using Mango (http://ric.uthscsa.edu/mango/) to visualise two-dimensional slices and MRIcroGL (https://www.nitrc.org/projects/mricrogl) to render a three-dimensional image.

In the first ALE, we tested for decreased brain activation in cancer patients treated with chemotherapy compared to healthy controls (CTx+ < HC). The second ALE examined increased brain activation in cancer patients treated with chemotherapy relative to healthy controls (CTx+ > HC). The third ALE tested for decreased brain activation in cancer patients treated with chemotherapy compared to no chemotherapy controls (CTx+ < CTx−). In the fourth ALE, we looked for increased brain activation in cancer patients treated with chemotherapy compared to no chemotherapy controls (CTx+ > CTx−). Finally, for contrasts that reported no significant clusters, we conducted an exploratory analysis, using an uncorrected *p* < .001 and minimum cluster sizes of 50mm^3^.

### Behavioral metrics

To investigate the second research question, correlation analyses from each study were reviewed to observe overall data trends. Next, we calculated effect sizes for main findings where information was available (i.e., *R*^*2*^ = squared correlation coefficient). Following Cohen’s (1988) conventions, .01 was regarded as a weak relationship, .09 a moderate relationship, and .25 a strong relationship.

## Results

### Search results

**Fig. 2:**
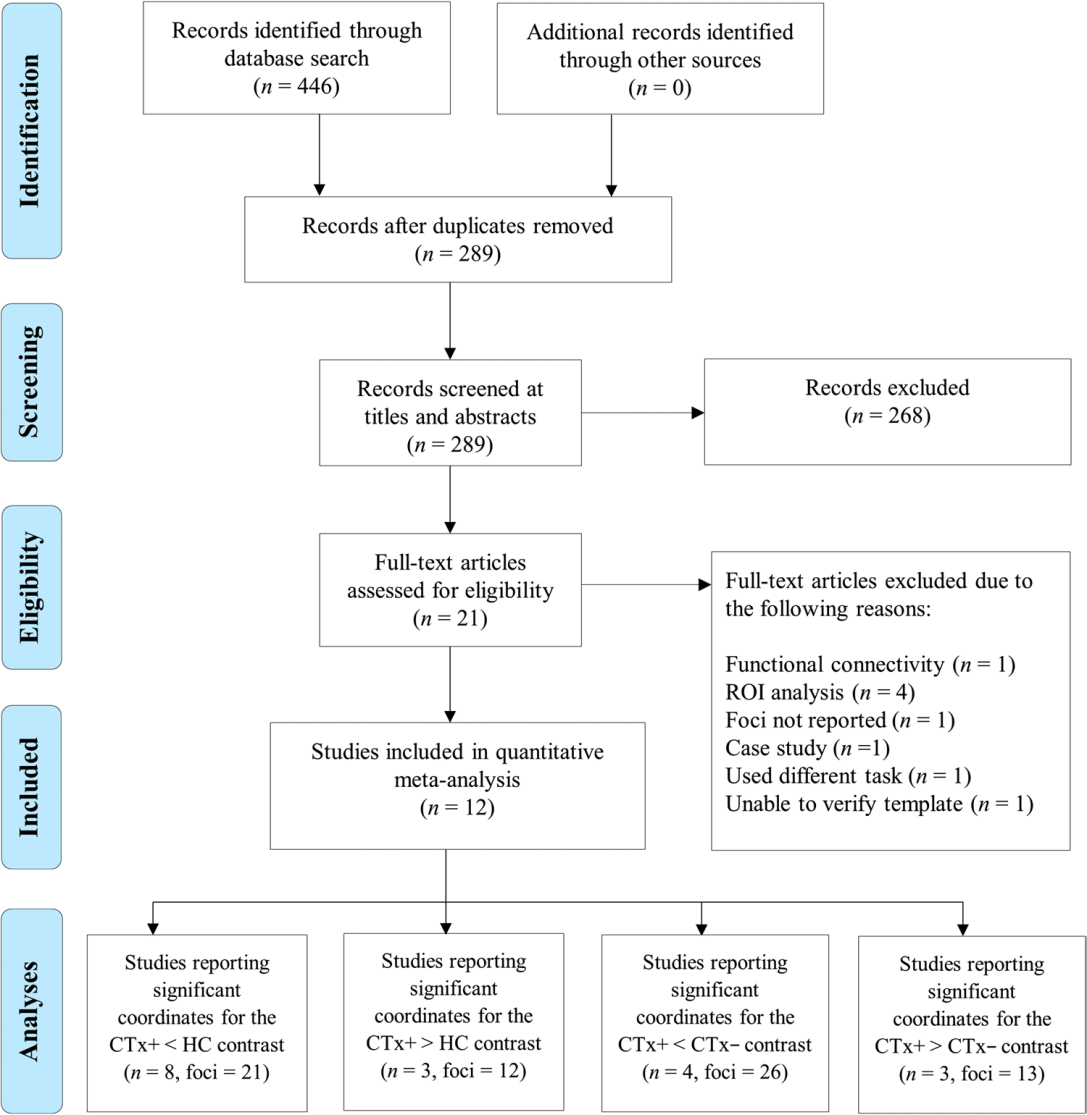
PRISMA flow diagram of the study selection process, adapted from Moher et al. (2009). *Note*. The final analysis section of the flow diagram depicts the total number of studies reporting significant coordinates for each group contrast and the total number of coordinates extracted from those studies. CTx+ = cancer patients treated with chemotherapy; CTx− = no chemotherapy controls; HC = healthy controls; < = decreased brain activation; > = increased brain activation; ROI = regions of interest; *n* = number of studies

The search of databases identified a total of 446 studies, of which 21 were assessed for eligibility at full text (see Fig. 2 for the PRISMA flow diagram). We excluded four studies, which conducted a region of interest (ROI) analysis (executive control subnetwork; Askren et al., 2014; Hosseini & Kesler, 2014; Jung et al., 2017; multitasking subnetwork; Deprez et al., 2014). We also omitted one case study (Ferguson et al., 2007), one resting-state fMRI study (Apple et al., 2018) and one study that used a facial expression fMRI task (Stouten-Kemperman et al., 2018). Moreover, one study was removed as we were unable to verify the brain template (Kam et al., 2016). Another study was excluded as the peak coordinates were not reported (Conroy, McDonald, Ahles, et al., 2013b). This resulted in a final set of 12 task-related fMRI studies published between 2009 and 2019, included in the current study. Of these 12 studies, eight reported significant results for the CTx+ < HC contrast (21 foci), three for the CTx+ > HC contrast (12 foci), four for the CTx+ < CTx− contrast (26 foci) and three for the CTx+ > CTx− contrast (13 foci; as can be seen in Fig. 2).

### Study characteristics

Studies were conducted in the United States of America (*n* = 7), Netherlands (*n* = 3) and Canada (*n* = 2). Cancer populations investigated included breast (10, 83%), ovarian (1, 8%) and colorectal, Hodgkin’s lymphoma, leukaemia and melanoma (1, 8%) cancers. The selected studies involved cancer patients treated with chemotherapy (*N* = 240), healthy controls (*N* = 103) and no chemotherapy controls (*N* = 134), with an average age of 53.72 years (*SD* = 7.7). Two studies used both a healthy control group and no chemotherapy control group. Four studies used a no chemotherapy control group and six studies used only a healthy control group. Of note, no chemotherapy controls encompassed cancer patients who had undergone surgery and, in some cases, had also received endocrine and/or radiation therapy as part of their cancer treatment (see Appendix 2). Most cancer patients were treated with standard-dose chemotherapy regimens, which included a combination of chemotherapeutic agents, such as cyclophosphamide, cisplatin, carboplatin, docetaxel, doxorubicin, epirubicin, fluorouracil, paclitaxel, methotrexate, and thiotepa. The time between completion of chemotherapy treatment and fMRI scan, spanned one month to 13 years. Various fMRI paradigms were utilised to evaluate brain activation including a wide array of working memory, executive function, episodic or verbal memory and attention tasks. Full clinical population characteristics and neuroimaging details are summarised in Appendix 2 Table 4 and Table 1, respectively.

### ALE meta-analyses

#### Alterations in cancer patients treated with chemotherapy compared to healthy controls.

No significant clusters were found between CTx+ patients and HC, using an exploratory uncorrected threshold (*p* < .001).

#### Alterations in cancer patients treated with chemotherapy compared to no chemotherapy controls

**Fig. 3:**
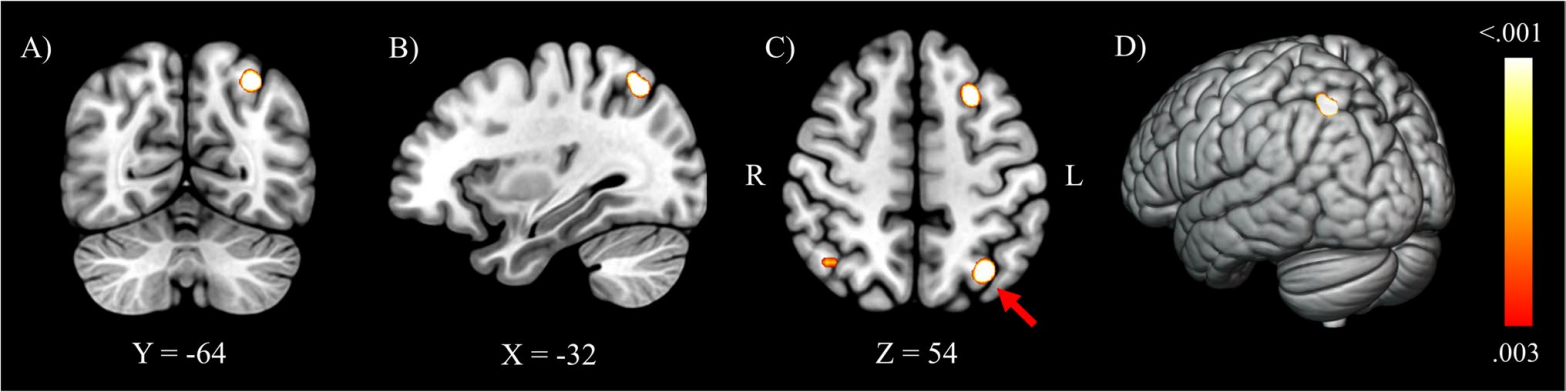
Meta-analytical maps of decreased brain activation in cancer patients treated with chemotherapy compared to no chemotherapy controls. *Note*: Decreased brain activation in the chemotherapy-treated group was localised in the left superior parietal lobe/precuneus displayed on coronal (A), sagittal (B) and axial (C) planes and a rendered image (D). Right and left hemispheres of the brain are presented according to radiological conventions. Values indicate MNI coordinates (x, y, z). Image thresholded at *p* < .003 uncorrected, for visualisation purposes. The largest cluster on the axial plane, indicated by a red arrow, is the only cluster that survived family-wise error corrections

As can be seen in Fig. 3, CTx+ patients showed a pattern of decreased brain activation in the left superior parietal lobe/precuneus (Brodmann area 7) compared to CTx− patients (FWE corrected). Table 2 provides the MNI coordinates of significant functional brain activation. No significant results were found in the opposite direction (CTx+ > CTx−) using an uncorrected threshold.

**Table 2:** Results from the ALE analysis of decreased brain activation in cancer patients treated with chemotherapy compared to no chemotherapy controls

			MNI coordinates				
Contrast	Cluster location	Size mm^3^	x	y	z	*N*	Sources
CTx+ < CTx−	Left superior parietal lobe/precuneus	736	−31	−63	52	2 1	de Ruiter et al. (2011) Stouten-Kemperman et al. (2015)

*Note.* Analyses were conducted using a cluster-level family-wise error threshold of *p* < .05, following an initial cluster-forming threshold of *p* < .001. CTx+ < CTx− = decreased brain activation in cancer patients treated with chemotherapy compared to no chemotherapy controls; MNI = Montreal Neurological Institute; *N* = number of foci of studies contributing to significant cluster

### Correlation between functional brain alterations and cognition

Seven of 12 studies tested for correlations between altered brain activation patterns and cognitive performance in the CTx+ group (Conroy, McDonald, Smith, et al., 2013a; Correa et al., 2017; de Ruiter et al., 2011; Kesler et al., 2009; Kesler et al., 2011; Stouten-Kemperman et al., 2015; Wang et al., 2016). Five of these seven studies (71%) reported a non-significant association between functional brain alterations and cognitive performance. Only two of these seven studies revealed a statistically-significant association (de Ruiter et al., 2011; Stouten-Kemperman et al., 2015). Specifically, reduced activation in the dorsolateral prefrontal cortex and hippocampal regions were related to a decline in cognitive performance on executive function (*R*^*2*^ = 59%) and episodic memory (*R*^*2*^ = 62%) tasks, respectively (de Ruiter et al., 2011; Stouten-Kemperman et al., 2015). These effect sizes were calculated from de Ruiter et al. (2011) only, as limited information was provided in Stouten-Kemperman et al. (2015). Following Cohen’s (1988) conventions, these associations can be regarded as strong relationships.

## Discussion

This study set out to map the common regions of altered brain activation patterns in non-CNS cancer patients who have undergone chemotherapy. By combining coordinates of peak activation from 240 cancer patients treated with chemotherapy (CTx+), 103 healthy controls (HC) and 134 no chemotherapy controls (CTx−), the present ALE meta-analysis is one of the first studies to quantitatively summarise the existing fMRI data. Our findings revealed decreased activation in the left precuneus located in the superior parietal lobe in CTx+ patients. No significant clusters were detected in three of our contrasts (i.e., CTx+ > CTx−, CTx+ < HC and CTx+ > HC). Furthermore, our results showed that the majority of studies did not provide evidence to suggest that altered brain activation following chemotherapy is related to performance on cognitive tasks. To this end, we will discuss a few methodological considerations for future task-related fMRI studies.

### The role of the precuneus in cancer patients treated with chemotherapy

Cancer patients who received chemotherapy showed reduced activation in the left precuneus, a region thought to play a pivotal role in a wide spectrum of highly integrated tasks, including episodic memory retrieval, visuospatial imagery and self-referential processing (Cavanna & Trimble, 2006). This data aligns with results from a previous ALE analysis by Kesler (2014), which revealed decreased activation in the precuneus of CTx+ patients. Findings are also consistent with a number of studies utilising resting-state fMRI (Chen et al., 2019; Simó et al., 2018), where decreased functional connectivity was observed in the left precuneus in CTx+ patients compared to healthy controls. However, our finding suggests that the alteration in the left precuneus may be a chemotherapy-specific effect. This is supported by the fact that our finding was identified in the contrast with cancer patients who had not received chemotherapy as a control group, hence accounting for variables that may arise from a cancer diagnosis (e.g., anxiety and depression) and surgery (e.g., post-operative dysfunction and inflammation; Li & Caeyenberghs, 2018). Cancer patients from the two studies, which reported coordinates of significant reduction in brain activation within the precuneus (de Ruiter et al., 2011; Stouten-Kemperman et al., 2015), were treated with a combination of fluorouracil, epirubicin and cyclophosphamide chemotherapeutic drugs. Although we cannot disentangle the specific cellular and molecular mechanisms underlying this potential brain alteration, these agents have been found in animal models to permeate the blood-brain-barrier and attack neurons in the CNS through mechanisms involving increased oxidative stress, demyelination and mitochondria disruption (for a review see Lv et al., 2020).

The precuneus may be particularly vulnerable to the neurotoxicity of chemotherapy drugs, given its high metabolic demands. The precuneus is a vastly connected hub that is part of a broader subnetwork (i.e., the default mode network; Utevsky et al., 2014). Due to its widespread connections with associated cortical and subcortical regions, the precuneus is considered functionally valuable for integrative processes (Cavanna & Trimble, 2006). Recent studies leveraging functional connectomes have also found that hub regions have higher metabolic demands and longer-distance connections compared to other brain regions, and could therefore be considered biologically costly (Crossley et al., 2014; Gollo et al., 2018). This high metabolic cost has been revealed in a wide array of clinical populations (Klaassens et al., 2017; Raizman et al., 2020), implicating the precuneus as a region that is often vulnerable to disease and ageing processes. Due to its high metabolic demand, it is possible that the precuneus receives more exposure to chemotherapy neurotoxicity, increasing susceptibility to direct damage and/or indirect disruptions to metabolic resources (Kesler & Blayney, 2016; Mounier et al., 2020). Further, chemotherapy mechanisms may exacerbate physiological cascades that are already impacted by ageing and disease, especially given the sample from our significant result were older cancer survivors (age > 56 years). With these considerations in mind, we provide novel evidence of chemotherapy-induced alterations in the left precuneus which may contribute to cognitive deficits in cancer patients.

Interestingly we did not find other brain regions of significant convergence across the 12 included studies. In particular, the lack of significant findings for the increased contrasts (CTx+ > HC; CTx+ > CTx–) was against expectations. At closer inspection, only 25 foci of significant increases in brain activation in CTx+ compared to controls (across 5 individual studies) in our ALE meta-analysis could be identified. The low number of foci can partially explain the non-significant findings of our meta-analysis. Similarly, our ALE meta-analysis did not reveal significant findings for the CTx+ < HC contrast, possibly due to the low number of areas of significant decreases in CTx + patients compared to healthy controls (21 foci, across 8 individual studies). The ALE method combines significant activation coordinates across multiple studies to uncover which brain regions are most frequently implicated in the literature (Eickhoff et al., 2012: Eickhoff et al., 2009). Moreover, the non-significant findings suggest that the brain regions, which revealed differences in activation in the individual studies, stem from widespread brain regions and therefore the meta-analysis cannot detect significant regions of convergence.

In addition, heterogeneity in the samples across the included studies may have hindered convergence in our meta-analysis (Muller et al., 2018). It has been suggested that functional brain alterations may follow a pattern which progresses over time, with increased compensatory brain activation occurring shortly after chemotherapy cessation and normalising over time, and decreased brain activation persisting over time (Koppelmans et al., 2012; Simó et al., 2013; Sousa et al., 2020). For example, the time between completing chemotherapy and fMRI scan varied considerably among studies. Five studies examined the acute effects of chemotherapy (i.e., < six months post-treatment) of which nearly half found increased cortical activation in CTx+ patients (2 studies, 40%; Kesler et al., 2009; Pergolizzi et al., 2019). Our finding of reduced activation in the precuneus in cancer patients who received chemotherapy compared to no chemotherapy controls was sourced from two studies (de Ruiter et al., 2011; Stouten-Kemperman et al., 2015), which tested cancer survivors in the chronic stage (i.e., > 10 years post-treatment). We suggest that increased brain activation in cortical regions could not be observed due to the combined acute and chronic effects of chemotherapy examined. To this end, convergence of activation in the precuneus in the CTx+ < CTx– contrast may have been found due to a number of communalities across the two studies (from the same research institutes) reporting this finding, including the use of similar inclusion/exclusion selection criteria for cancer patients (e.g., the type of chemotherapeutic agent), the fMRI task (i.e., episodic memory) and scanning protocols (de Ruiter et al., 2011; Stouten-Kemperman et al., 2015).

### Correlation between functional brain alterations and cognition

A review of the fMRI literature demonstrated that the majority of previous studies (5/7) did not find an association between altered brain activation patterns and cognitive performance in CTx+ patients. Only two of these studies reported a significant relationship and only one study (de Ruiter et al., 2011) provided sufficient information to compute an effect size. Specifically, we found a strong correlation between altered brain activation and cognitive functioning, such that increased pathology predicts poorer performance on cognitive tasks (i.e., executive function and episodic memory) following chemotherapy. Despite the limited evidence, several functional neuroimaging studies have characterised brain function profiles as ‘biomarkers’ of cognitive functioning (e.g., Kesler et al., 2011). We suggest that these interpretations may be premature and future research examining the relationship between brain-based measures and detailed cognitive assessments is warranted.

### Limitations and future directions

There are three notable limitations that impact interpretation of the current results. First is the relatively small number of task-related fMRI studies that have been published. The cluster of reduced activation in the precuneus was found from two of four studies in the CTx+ < CTx− contrast (Table 2). Considering Eickhoff et al. (2016) recommends around 17–20 studies in each dataset to obtain robust results, our ALE analyses may not hold sufficient power to detect small effects, partial out subject-specific variation and ensure that results are not led by single experiments (Muller et al., 2018). Nonetheless, our meta-analysis has combined the most fMRI data on this topic to date. To consolidate findings and clarify other neural abnormalities in CTx+ patients, additional task-related fMRI studies need to be conducted to run a comprehensive meta-analysis on the same topic with a larger sample (e.g., 20 studies) in the future.

The second limitation of the present study is the variation in clinical sample characteristics across the selected fMRI studies (as can be seen in Appendix 2). First, the mix of chemotherapeutic drug combinations may have resulted in fewer robust patterns. Animal models have shown that some agents are more neurotoxic than others (Christie et al., 2012; Yan et al., 2015). For example, studies included a combination of blood-brain-barrier permeable (e.g., methotrexate, fluorouracil, cyclophosphamide) and impermeable chemotherapeutic agents (e.g., doxorubicin), which can induce mixed effects on the CNS (Carozzi et al., 2015). Second, as raised above, the variability in post-chemotherapy time intervals may facilitate divergent brain activation patterns. With the studies ranging from over one-month (Pergolizzi et al., 2019) to 13 years (Stouten-Kemperman et al., 2015) after chemotherapy, it is possible that a mixture of transient compensatory mechanisms and long-term reduced brain alterations are present within this literature. The current number of available studies is too small to separate data according to the abovementioned variables. As the field progresses and more studies are published, we can pool data based on these clinical subgroups and consequently, discover further patterns of altered brain activation in CTx+ patients.

The third limitation of our study is that analyses may be hampered by inconsistencies across the methodological features of the included studies (as can be seen in Table 1). First, the fMRI tasks utilised varied across studies; they comprised an assortment of executive function (i.e., Tower of London), memory (i.e., paired associates learning and levels of processing) and attention (i.e., n-back) tasks. This is a relevant issue, as tasks belonging to different neuropsychological domains activate different brain regions (Simó et al., 2013). Future research would benefit from employing fMRI tasks from the Human Connectome Project dataset (Barch et al., 2013), which have been well-validated and demonstrate reliable activation in function-specific regions, producing data amenable to concatenation. Second, the results of ALE meta-analyses are guided by statistically significant regions identified in previous studies in the form of stereotaxic coordinates (Eickhoff et al., 2009; Eickhoff et al., 2012). The probability of the activity of a voxel or cluster differing between groups is largely affected by the choice of statistical threshold applied. In our meta-analysis, 58% of studies employed low uncorrected thresholds. Studies using less stringent statistical thresholds are more likely to report findings that are significant than studies with stricter thresholds (Acar et al., 2018). This undoubtedly explains some of the variability observed in the literature on functional brain alterations in cancer patients. While ALE is able to remove some between-study variability (i.e., sample size), the fact that the analysis exclusively combines data for statistically significant sites only, suggests it may introduce bias (Acar et al., 2018). Future researchers should take this bias into account by applying cluster-level family-wise error correction (*p* < .05), as this thresholding procedure has low susceptibility to false positives (Muller et al., 2018).

Despite these limitations, patterns of abnormal brain activation are emerging from task-related fMRI findings on cancer survivors. A strength of the current study lies in the ALE approach used to collate data across diverse neuroimaging studies to clarify known inconsistencies (Sousa et al., 2020). We also performed the most up-to-date literature search of task-related fMRI data on cognitive deficits in cancer patients. For example, compared to a previous ALE analysis (Kesler, 2014), we included six additional studies that were not previously incorporated (Correa et al., 2017; Menning et al., 2017; Pergolizzi et al., 2019; Stouten-Kemperman et al., 2015; Vardy et al., 2019; Wang et al., 2016).

## Conclusion

The present ALE meta-analysis is one of the first to combine a series of recent task-related fMRI studies to identify reliable regions of altered brain activation patterns in CTx+ patients. After accounting for cancer-related variables, we found a pattern of reduced activation in the left precuneus of CTx+ patients. Our results provide insight into a possible chemotherapy-induced alteration in cancer survivors, potentially guiding targeted treatment strategies. Further studies are needed to perform a larger-scale meta-analysis using harmonised task-related fMRI protocols, evaluating the effects of chemotherapeutic agents and post-chemotherapy time intervals on brain activation patterns. With continued investigation, fMRI may be considered a useful biomarker of cognitive deficits in the future.

## Data Availability

The data can be made available upon request to the corresponding author.

## Appendix 1 Full Search Syntax Used in Systematic Review

**Table 3:** Subject headings and search string used on titles and abstracts for the ALE meta-analysis

fMRI	Cancer	Chemotherapy	Cognitive dysfunction
functional magnetic resonance, functional imaging, functional MRI, fMRI, BOLD, blood oxygen* level dependent, brain activation	cancer, neoplasm, tumour, tumor, malignancy, patient, survivor	chemotherapy, chemotherapeutic agent*, chemo*, ctx, adjuvant therapy, adjuvant treatment, adjunctive therapy	chemobrain, chemofog, chemotherapy induced cognitive changes, CICI, chemotherapy related cognitive changes, CRCI, neuropsychol*, cognit, memory, attention, executive function, concentrat*, language, verbal, learning

*Note.* The search strings were combined using the Boolean operators AND and OR

## Appendix 2 Clinical Population Characteristics

**Table 4:** Overview of clinical population characteristics from the included fMRI studies

Study	*N* and sex of participants		Mean age (standard deviation) in years		Stage and type of cancer	Months since chemotherapy	Chemotherapy regimens	Adjuvant treatment
	CTx+	Control	CTx+	Control				
Conroy, McDonald, Smith, et al. (2013a)	24F	23F^a^	57.8 (9.6)	61.2 (9.9)^a^	Stage 1–3 breast cancer	77	AC, ACT, CAF, CMF or other	Radiation therapy, tamoxifen and/or aromatase inhibitor
Correa et al. (2017)	17F	18F^a^	56.1(8.2)	56.2 (9.1)^a^	Stage 1–4 ovarian, fallopian tube or peritoneal cancer	3	Average 6 cycles with CP, PC or PC-carboplatin	Abdominal hysterectomy and bilateral salpingo-oophorectomy surgery
de Ruiter et al. (2011)	16F	15F^b^	56.3 (5.5)	58.2 (5.8)^b^	High-risk breast cancer for CTx+. Stage 1 breast cancer for CTx− group	114	4 cycles of FEC, followed by 1 cycle of high-dose CTC	Tamoxifen for CTx+. Local surgery and locoregional radiation therapy for both cancer groups
Kesler et al. (2009)	14F	14F^a^	55.1 (8.0)	54.2 (8.0)^a^	Stage 3–4 breast cancer	3	6 cycles of CMF or 4–8 cycles of ACT	Radiation therapy and tamoxifen
Kesler et al. (2011)	22F	17F^a^ 18F^b^	56.2 (7.8)	58.1 (6.5)^a^ 55.6 (9.4)^b^	Stage 2–3 breast cancer for CTx+. Stage 0–2, breast cancer for CTx−	56	ACT or without adriamycin, AC, CMF or other	Radiation therapy and tamoxifen for both cancer groups
Lopez Zunini et al. (2013)	21F	21F^a^	50.6 (8.4)	49.7 (8.8)^a^	Stage 1–3 breast cancer	1	5–7 cycles of FEC-docetaxel, 4 cycles CD or other	Lumpectomy or mastectomy surgery
McDonald et al. (2012)	16F	15F^a^ 12F^b^	52.9 (8.6)	50.5 (6.0)^a^ 52.7 (7.2)^b^	Stage 1–3 breast cancer for CTx+. Stage 0–2 breast cancer stage for CTx−	12	ACT, TAC or AC	Radiation therapy, antiestrogen therapy and psychotropic medications for both cancer groups
Menning et al. (2017)	24F	20F^b^	49.4 (8.8)	51.2 (6.8)^b^	Stage 1–3 breast cancer for CTx+. Stage 0–1 for CTx−	6	3–6 cycles of AC-texane, 4–6 cycles of AC or 3–6 cycles of FEC	Radiation therapy for both cancer groups, but tamoxifen for CTx+ only
Pergolizzi et al. (2019)	13F	12F^a^	47.9(6.4)	48.5 (8.0)^a^	Stage 1–3 breast cancer	1	ACT or CMF	Surgical resection
Stouten-Kemperman et al. (2015)	24F	15F^b^	59.8 (6.3)	58.2 (5.8)^b^	Stage 1–4 breast cancer for CTx+. Stage 1 breast cancer for CTx−	161	5 cycles of FEC	Radiation therapy and tamoxifen for CTx+, radiation therapy for CTx-. surgery for both cancer groups
Vardy et al. (2019)	34F	23F ^b^	*48.4 (30–60)	*54.10^b^ (30–59)	Stage 3 breast cancer for both CTx+ and CTx−		Chemotherapy regimen not reported	Radiation therapy, endocrine treatment and lumpectomy or mastectomy surgery for both cancer groups
Wang et al. (2016)	2M 13F	1M^a^ 13F	50.7 (7.5)	53.0 (7.2)^a^	Stage 1–4 breast cancer, colorectal cancer, Hodgkin’s lymphoma, leukemia or myeloma	< 6	Chemotherapy regimen not reported, >3 cycles	Adjuvant treatment not reported

*Note.* CTx+ = cancer patients treated with chemotherapy; CTx− = no chemotherapy controls, F = females, M = males, * = median age (range). Chemotherapeutic drug combinations include: AC = doxorubicin, cyclophosphamide; ACT = doxorubicin, cyclophosphamide, paclitaxel/docetaxel; CAF = cyclophosphamide, doxorubicin, fluorouracil; CMF cyclophosphamide, methotrexate, fluorouracil; CD = carboplatin and docetaxel; CP = carboplatin, paclitaxel; CTC = carboplatin, thiotepa, cyclophosphamide; FEC = fluorouracil, epirubicin, cyclophosphamide; PC = paclitaxel, cisplatin; TAC = docetaxel, doxorubicin, cyclophosphamide

^a^No chemotherapy controls. ^b^ Healthy controls
